# Comparative Evaluation of White Quinoa and Tri‐Color Quinoa on Indomethacin‐Induced Gastrointestinal Damage

**DOI:** 10.1002/fsn3.72418

**Published:** 2026-09-30

**Authors:** Nasser S. Alqahtani, Hanaa M. Hassan, Hanaa M. Mohamed, Fawzia M. Gazaly, Ragaa A. Sadeek

**Affiliations:** ^1^ Community Health Department Northern Border University Arar Saudi Arabia; ^2^ Department of Agricultural Chemistry, Faculty of Agriculture Minia University El‐Minia Egypt; ^3^ Department of Home Economics, Faculty of Specific Education Minia University El‐Minia Egypt

**Keywords:** histopathological, necrosis, NSAIDs, pseudocereal, submucosal

## Abstract

Nonsteroidal anti‐inflammatory drugs (NSAIDs), such as indomethacin, frequently cause gastric injury, often characterized by ulceration. This study investigated the comparative gastroprotective effects of white quinoa and tri‐color quinoa (a combination of red, black, and white varieties) in mitigating indomethacin‐induced gastric damage in male rats. Thirty‐six male rats were divided into dietary groups and administered quinoa‐supplemented diets for 21 days before gastric injury was induced using indomethacin. Biochemical markers, including TNF‐α and IL‐6, were assessed, and molecular docking was employed to examine the interactions of quinoa‐derived compounds, such as procyanidin B2 and cinchonain IB, with target proteins Atp4a, COX‐2, and NF‐κB. Tri‐color quinoa demonstrated greater efficacy than white quinoa, reducing mucosal necrosis by 83%, attenuating oxidative stress, and significantly enhancing anti‐inflammatory markers (*p* < 0.05). Furthermore, bioactive compounds showed strong binding affinities (−7.1 to −8.9 kcal/mol) to target proteins through hydrogen and hydrophobic interactions. Histological examination confirmed superior protection for the tri‐color group, displaying near‐normal mucosa (lesion score 0.4 vs. 2.4) and minimal submucosal edema. In conclusion, tri‐color quinoa provides more effective protection against NSAID‐induced gastric injury than white quinoa, likely due to its enhanced phytochemical profile and broader range of plant‐derived compounds that collectively reduce inflammation and preserve gastric mucosal integrity.

## Graphical abstract

This graphical abstract presents a comparative study on the gastroprotective efficacy of white versus tri‐color quinoa in male rats against indomethacin‐induced gastric injury. Following a 21‐day dietary intervention with either white or tri‐color quinoa, indomethacin was administered to induce gastric damage. Molecular docking analysis revealed strong binding affinities between quinoa‐derived bioactive compounds, such as procyanidin B2, and target proteins including Atp4a, NF‐κB, and COX‐2. The results demonstrated that tri‐color quinoa provided significantly superior protection, reducing mucosal necrosis by 83%, significantly lowering pro‐inflammatory markers (TNF‐α and IL‐6, *p* < 0.05), and preserving a near‐normal gastric mucosa (with a lesion score of 0.4 compared to 2.4 for the white quinoa group). This enhanced gastroprotection is attributed to the richer phytochemical profile and synergistic activity within the tri‐color variety.

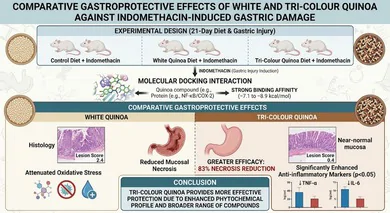

## 1 Introduction

Although nonsteroidal anti‐inflammatory drugs (NSAIDs) are essential tools for managing pain, fever, and inflammation, they are notorious for causing significant gastrointestinal complications. This toxicity arises primarily because these agents suppress the biosynthesis of gastro protective prostaglandins by inhibiting both COX‐1 and COX‐2 isoforms. Although novel approaches, including NO‐NSAIDs and dual‐acting COX/LOX inhibitors, aim to mitigate these risks, their comparative safety and effectiveness are not yet fully established. Beyond prostaglandin suppression, NSAIDs activate damaging inflammatory pathways by elevating levels of pro‐inflammatory cytokines, such as tumor necrosis factor‐alpha (TNFα) and interleukin‐6 (IL‐6), which further accelerates oxidative stress, gastric mucosal injury, and deep ulceration (Sohail et al. 2023; Zheng et al. 2025).

Conventional gastroprotective therapies, including proton pump inhibitors (PPIs) and H_2_‐receptor antagonists, are commonly employed to manage these side effects; however, their long‐term administration is increasingly associated with significant clinical limitations. Recent evidence highlights concerns regarding potential adverse outcomes, including nutrient malabsorption, increased vulnerability to infections, chronic kidney disease, osteoporosis‐related fractures, and gut dysbiosis (Feng et al. 2021; Maideen 2023). These constraints emphasize the urgent need for safer, natural nutraceutical alternatives that provide effective mucosal protection with a more favorable safety profile.

Consequently, there is a paradigm shift toward exploring natural functional foods with potent inherent antioxidant and anti‐inflammatory properties (González‐Montoya et al. 2018). Quinoa ( *Chenopodium quinoa* Willd.), a climate‐resilient pseudocereal, has emerged as a promising anti‐ulcerogenic candidate owing to its exceptional and diverse phytochemical profile (Vega‐Gálvez et al. 2010; Tang et al. 2015). Crucially, the therapeutic potential of quinoa is driven by specific, highly bioavailable bioactive compounds that possess targeted cytoprotective properties. Among these, procyanidin B2, a powerful oligomeric proanthocyanidin, has been reported to exhibit superior ability to scavenge reactive oxygen species (ROS), inhibit lipid peroxidation, and suppress NF‐κB activation, thereby preventing inflammatory tissue degradation. Concurrently, cinchonain IB, a complex flavanol derivative found in quinoa, has been associated with anti‐inflammatory and tissue‐healing effects by modulating cytokine networks and reinforcing cellular defense mechanisms. Furthermore, flavonols such as kaempferol‐3‐O‐rutinoside may contribute to downregulating inducible nitric oxide synthase (iNOS) and COX‐2 expressions, while enhancing the gastric mucus barrier and maintaining intracellular antioxidant enzymes (Gaobotse et al. 2023). Importantly, this phytochemical density varies drastically by variety. Colored types, such as red and black quinoa, typically exhibit significantly higher total phenolic content and greater radical scavenging capacities compared to the traditional white variety (Miranda et al. 2010).

While previous studies have reported the general gastro protective potential of quinoa extracts (Paśko et al. 2010), a direct comparative evaluation between white quinoa (WQ) and tri‐color quinoa (TCQ; a formulation combining red, black, and white varieties) against NSAID‐induced gastric injury has not yet been conducted. Understanding how these varietal differences and specific phytochemical matrices correlate with gastro protective efficacy is vital for designing evidence‐based dietary interventions to manage NSAID‐induced gastropathy.

To unravel these mechanisms at a molecular level, the core regulatory targets of gastric homeostasis must be considered. Atp4a (the gastric proton pump) represents a critical target for evaluating potential acid‐suppressing activity, while COX‐2 and NF‐κB represent the cornerstone pathways mediating the inflammatory response and mucosal injury during ulcer formation. Therefore, this study aims to evaluate and compare the protective effects of WQ and TCQ against indomethacin‐induced gastric injury in a rat model, integrated with a molecular docking assessment of quinoa's key leads (procyanidin B2, cinchonain IB, and kaempferol‐3‐O‐rutinoside) against Atp4a, COX‐2, and NF‐κB. We centrally hypothesize that TCQ, owing to its broader and superior phytochemical diversity, will exert enhanced gastro protective efficacy compared to WQ, directly correlating with a superior capacity to modulate inflammatory signaling (TNFα—IL‐6), attenuate oxidative stress, and preserve histopathological mucosal integrity.

## 2 Methodology

### 2.1 Source of Samples

Organic whole‐grain white quinoa ( *Chenopodium quinoa* Willd.) and tri‐color quinoa (a uniform mixture of red, black, and white varieties) were obtained as whole seeds from Organic Nation Market (Cairo, Egypt; Origin: Peru; Brand: Organic Nation). To ensure complete compositional and phytochemical consistency throughout the study, all quinoa grains for each respective variety were sourced from the same production batch (Batch No. OQN‐2024‐05) and freshly ground into fine flour prior to diet preparation. Refined wheat flour (72% extraction rate), baker's yeast ( *Saccharomyces cerevisiae* ), granulated sugar, unsalted butter, iodised salt, and pasteurized milk were also sourced from local commercial suppliers. All ingredients were of certified food‐grade quality and stored under standard food safety conditions (dry, dark, and pest‐free) until use (Kanimozhi and Arivuchudar 2023).

### 2.2 Preparation of Experimental Quinoa‐Based Diets

The experimental diets were formulated by incorporating functional bread into a standard basal rodent diet. The bread was prepared using a standardized recipe to ensure consistency across all groups. For every 1000 g of dough, the following ingredients were utilized:

- Flour (500 g): Either refined wheat flour (for C^−^ and C^+^ groups), white quinoa flour (for WQ groups), or tri‐color quinoa flour (for TCQ groups).
- Distilled water (300 mL): Added gradually to achieve optimal dough consistency.
- Additives: 10 g dry yeast, 5 g iodised salt, and small amounts of sugar and butter to ensure standard bread texture and palatability for the rats.

The preparation followed a systematic didactic procedure: (1) manual kneading for 15 min until a homogenous elastic dough was formed; (2) fermentation in a controlled environment at 37°C for 60 min; and (3) baking in a preheated electric oven at 180°C for 25 min. Once cooled, the bread was air‐dried, finely ground, and mixed with the standard basal diet at a ratio of 1:3 (25% bread to 75% standard diet). This specific ratio was chosen to provide a concentrated dose of quinoa's bioactive compounds while maintaining the caloric and nutritional balance required for the rats.

### 2.3 Animals

Thirty‐six male Sprague Dawley rats (mean body weight: 160.9 ± 2 g) were procured and acclimatized for 14 days at the Biological Laboratory, Faculty of Agriculture, Minia University. The animals were randomly assigned to six experimental groups (*n* = 6 rats per group). This sample size was strictly determined based on the ethical “3Rs” principle (Replacement, Reduction, and Refinement) to minimize animal use and suffering while maintaining sufficient statistical power to achieve significant differences (*p* < 0.05). To comply with animal welfare spatial requirements, each group of six rats was housed across two polypropylene cages (47 × 34 × 18 cm), with three rats kept per cage under controlled environmental conditions (temperature: 25°C ± 2°C; 12‐h light/dark cycle).

To ensure uniform exposure to the bioactive compounds, the animals were provided with controlled daily portions of the experimental diets administered as a homogeneous mash. The quinoa‐incorporated bread was processed into a fine powder and thoroughly blended with the pulverized basal diet (25:75 ratio) to achieve a consistent mixture, thereby preventing selective consumption. To rigorously monitor and evaluate dietary compliance, a predefined quantity of the fresh mash diet (75 g per cage; approximately 25 g per rat) was introduced daily. Every 24 h, the unconsumed food remnants and spillages from each cage were carefully collected, dried, and weighed. Daily food intake per rat was calculated by subtracting the weight of the remnants from the initial supplied amount and dividing by the number of rats per cage (*n* = 3). Based on these precise daily measurements, the actual food consumption was found to be statistically uniform across all experimental groups, fluctuating narrowly between 21 and 23 g per rat daily. Cumulative and average weekly food consumption was compared across all groups to ensure statistical uniformity and to firmly validate that the observed consistency in body weight was indeed a reflection of equitable and verified dietary intake. Daily health checks were performed, and body weight gain was recorded weekly to monitor the overall nutritional status and development of the animals across all experimental groups. Throughout the study, the rats had ad libitum access to filtered water. Animal handling and experimental procedures adhered to ethical guidelines approved by the Institutional Animal Ethics Committee of Minia University (Approval No: MU/FA 017/01/24) (Bancroft and Gamble 2008).

### 2.4 Chemicals and Reagents

Indomethacin (≥ 99% purity, CAS: 53‐86‐1) was purchased from Sigma‐Aldrich (Cairo, Egypt). The following Bio‐Diagnostics assay kits were used: pepsin activity (PE‐2510), gastric mucin (MU‐3015), reduced glutathione (GL‐4120), catalase (CA‐3718), malondialdehyde (MD‐3322), cyclooxygenase (COX‐4415), and NF‐κB (NF‐5210). Indomethacin was stored at 4°C, and assay kits were kept at −20°C until analysis (Ohkawa 1979).

### 2.5 Preparation of Quinoa Seed Powders

Quinoa seeds were manually cleaned, rinsed with cold water (4°C) to remove saponins (foam reduction), soaked for 6 h at 25°C, and shade‐dried to < 10% moisture content. Dried seeds were milled using a 0.5 mm sieve, yielding 94% flour (white quinoa) and 92% flour (tri‐color quinoa) per 100 g of seeds. The powders were stored in amber airtight containers at 4°C to maintain bioactivity (Pathan and Siddiqui 2022). For the phytochemical analysis, a precise quantity of 100 g of the prepared quinoa powder was extracted using 1000 mL of 80% aqueous ethanol (solid‐to‐solvent ratio of 1:10 w/v). The mixture was stirred for 24 h at room temperature, filtered through Whatman No. 1 paper, and concentrated under reduced pressure at 40°C using a rotary evaporator. The resulting crude extracts were stored at −20°C until further use according to Carciochi et al. (2015).

### 2.6 Flour Replacement Protocol

White quinoa powder (WQP) and tri‐color quinoa powder (TCQP) were each used to replace 40% of wheat flour (WF) in the experimental formulations, resulting in a blend of 60% WF and 40% quinoa powder (Table 1). The blended flours were packed in sealed polyethylene bags and stored at 4°C until bread preparation.

**TABLE 1:** Replacement formulation.

Abbreviation of sample	Replacement (by dry weight)
WF 100%	100% wheat flour
WQP 40%	60% wheat flour + 40% white quinoa powder
TQCP 40%	60% wheat flour + 40% tri‐color quinoa powder

*Note:* Based on Sharawy et al. (2023).

### 2.7 Bread Preparation Protocol

As part of formulating the experimental diets detailed in Section 2.2, bread was prepared using four different flour blends, following the method described by Sharawy et al. (2023) (Balaha et al. 2022), with minor modifications (Table 2). Ingredientsflour, butter, sugar, salt, and yeast were mixed with water or milk and manually kneaded for 5–10 min until a uniform dough was formed. The dough was then processed as follows:

1. Proofed for 55 min under covered conditions;
2. Shaped and rested for 30 min;
3. Baked at 180°C for 30 min.

**TABLE 2:** Bread ingredients.

Samples/Ingredients	WF 100%	MIX 20%	MIX 30%
WF (g)	440	352	308
FF	—	44	66
CHF (g)	—	44	66
Butter (g)	42	42	42
Salt (g)	6	6	6
Sugar (g)	22	22	22
Moist yeast (g)	7	7	7
Water (mL)	174	174	174
Milk (mL)	125	125	125

*Note:* MIX 20%: blend of 80% wheat flour and 20% quinoa flour fractions (10% FF + 10% CHF). MIX 30%: blend of 70% wheat flour and 30% quinoa flour fractions (15% FF + 15% CHF).

Abbreviations: CHF, coarse husk fraction (quinoa bran); FF, fine fraction (quinoa flour); WF, wheat flour.

After baking, the loaves were cooled and stored in polyethylene bags for subsequent analysis and incorporation into the respective experimental diets.

### 2.8 Experimental Design

Following a 1‐week acclimatization period, 36 male rats were randomly assigned to six groups (*n* = 6 per group). Each group received a distinct dietary treatment for 21 days, with unrestricted access to water and controlled food portions (Table 3; Bancroft and Gamble 2008).

**TABLE 3:** Diet and indomethacin conditions for each group.

C^−^ (negative control)	75% standard diet, 25% wheat bread; no indomethacin
C^+^ (positive control)	75% standard diet, 25% wheat bread; given indomethacin
WQ^−^	75% standard diet, 25% white quinoa bread; no indomethacin
TCQ^−^	75% standard diet, 25% tri‐color quinoa bread; no indomethacin
WQ^+^	75% standard diet, 25% white quinoa bread; given indomethacin
TCQ^+^	75% standard diet, 25% tri‐color quinoa bread; given indomethacin

### 2.9 Induction of Indomethacin‐Induced Gastric Injury

After the 21‐day feeding period, indomethacin‐treated groups (C^+^, WQ^+^, TCQ^+^) underwent a 24‐h fast (Gohar and Zaki 2014). Gastric injury was induced by oral administration of 30 mg/kg indomethacin in 1% carboxy methyl cellulose solution (Mizoguchi et al. 2001). Rats were euthanized under ether anesthesia 24 h post‐administration (Zhu and Yan 1996).

### 2.10 Gastric Content Analysis

Stomachs were excised following cardiac‐end ligation, rinsed with ice‐cold saline (0.9% NaCl), and blotted dry. Gastric contents were collected from the fundic region, centrifuged at 3500 rpm for 15 min at 4°C, and filtered through 0.45 μm membrane filters. Only samples containing ≤ 0.5 mL particulate matter were included in the analysis (Beutler et al. 1963).

### 2.11 Biomarker Assessment and Inflammatory Molecular Marker Analyses

Stomach tissues were homogenized in ice‐cold phosphate buffer (100 mM, pH 7.4) using a chilled glass homogenizer (Wheaton, Millville, NJ, USA). The homogenates were centrifuged at 7000 rpm for 20 min at 4°C using a cooling centrifuge (Sigma 3‐30KS, Oster ode am Harz, Germany). The resulting supernatants were collected and stored at −80°C for the following biochemical analyses.

#### 2.11.1 Oxidative Stress Markers

##### 2.11.1.1 MDA (Malondialdehyde)

Measured colorimetrically using a commercial assay kit (Bio‐Diagnostic, Giza, Egypt) based on the thiobarbituric acid (TBA) method. Briefly, TBA reacts with MDA in an acidic medium at 95°C for 30 min to form a pink reactive product, and the absorbance was measured at 534 nm according to the method of Ohkawa (1979).

##### 2.11.1.2 GSH (Reduced Glutathione)

Determined colorimetrically using a commercial assay kit (Bio‐Diagnostic, Giza, Egypt) based on the reduction of 5,5′‐dithiobis(2‐nitrobenzoic acid) (DTNB) with GSH to produce a yellow‐colored compound. The absorbance was measured at 405 nm according to the method of Beutler et al. (1963).

##### 2.11.1.3 CAT (Catalase Activity)

Evaluated colorimetrically using a commercial assay kit (Bio‐Diagnostic, Giza, Egypt) based on the formulation of Aebi (1984). Catalase reacts with a known quantity of H_2_O_2_, and the reaction is stopped after exactly 1 min with a catalase inhibitor. The remaining H_2_O_2_ reacts with 3,5‐dichloro‐2‐hydroxybenzenesulfonic acid (DHBS) and 4‐aminophenazone to form a chromophore, measured at 510 nm. All colorimetric measurements were performed using a UV–Visible Spectrophotometer (UV‐1800; Shimadzu, Kyoto, Japan).

#### 2.11.2 Inflammatory and Molecular Markers

To evaluate the anti‐inflammatory potential of the extracts and their capacity to modulate the inflammatory cascade triggered by the ulcerogenic agent, COX‐2 was selected as a pivotal inducible molecular target alongside NF‐κB.

##### 2.11.2.1 COX‐2 Expression

Quantified using a Rat COX‐2 solid‐phase sandwich ELISA kit (cat # ab210972; Abcam, Cambridge, UK) following the manufacturer's instructions. The optical density of the color complex was measured spectrophotometrically at 450 nm, and the concentration was determined relative to a standard curve.

##### 2.11.2.2 NF‐κB Activation

Assessed using a Nuclear NF‐κB p65 Transcription Factor Assay Kit (cat # ab133112; Abcam, Cambridge, UK). This assay utilizes a specific double‐stranded DNA sequence containing the NF‐κB response element immobilized on a 96‐well plate. Active NF‐κB from nuclear extracts specifically binds to this oligonucleotide and is detected using a specific primary anti‐NF‐κB p65 antibody and an HRP‐conjugated secondary antibody, with colorimetric quantification at 450 nm.

#### 2.11.3 Biochemical Analysis of Gastric Secretions (Atp4a, Pepsin, and Mucin)

To investigate whether the quinoa‐derived compounds possess an antisecretory effect capable of reducing acid aggressiveness, Atp4a (ATPase) was targeted as the final common pathway regulating gastric acid secretion, evaluated alongside other key gastric biochemical parameters (pepsin and mucin).

##### 2.11.3.1 Pepsin and Mucin Quantification

Gastric juice was collected from each rat and centrifuged at 3500 rpm for 10 min at 4°C to remove debris. Pepsin activity was determined according to Anson (1938). Briefly, 0.1 mL of gastric juice was incubated with 0.5 mL of 2% w/v bovine hemoglobin (in 0.06 N HCl) at 37°C for 20 min. The reaction was stopped with 10% TCA. After centrifugation, the absorbance of the supernatant was measured at 280 nm. A standard curve of tyrosine was used for quantification, and results were expressed as μEq of tyrosine released/mL.

For Mucin concentration, the Alcian blue binding assay (Corne et al. 1974) was employed. Approximately 100 mg of glandular gastric tissue segments were soaked in 0.1% Alcian blue solution for 2 h. Uncombined dye was removed by washing with 0.25 M sucrose. The mucin‐bound dye was eluted with 10 mL of 0.5 M magnesium chloride and shaken with an equal volume of diethyl ether. The absorbance was measured at 605 nm. Mucin content was expressed as μg of Alcian blue recovered per gram of wet tissue weight. To ensure reproducibility, all assays were performed in triplicate with an intra‐assay CV < 10%.

##### 2.11.3.2 Molecular Interaction Analysis

To support the biochemical findings regarding Atp4a, COX‐2, and NF‐kB modulation, the structural binding patterns and molecular interactions of the active quinoa compounds were mapped. As shown in Figure 1, the 3D and 2D interaction maps detail the hydrogen bonding, hydrophobic alkyl, and electrostatic forces driving the stability of the optimal ligand–protein complexes.

**FIGURE 1:**
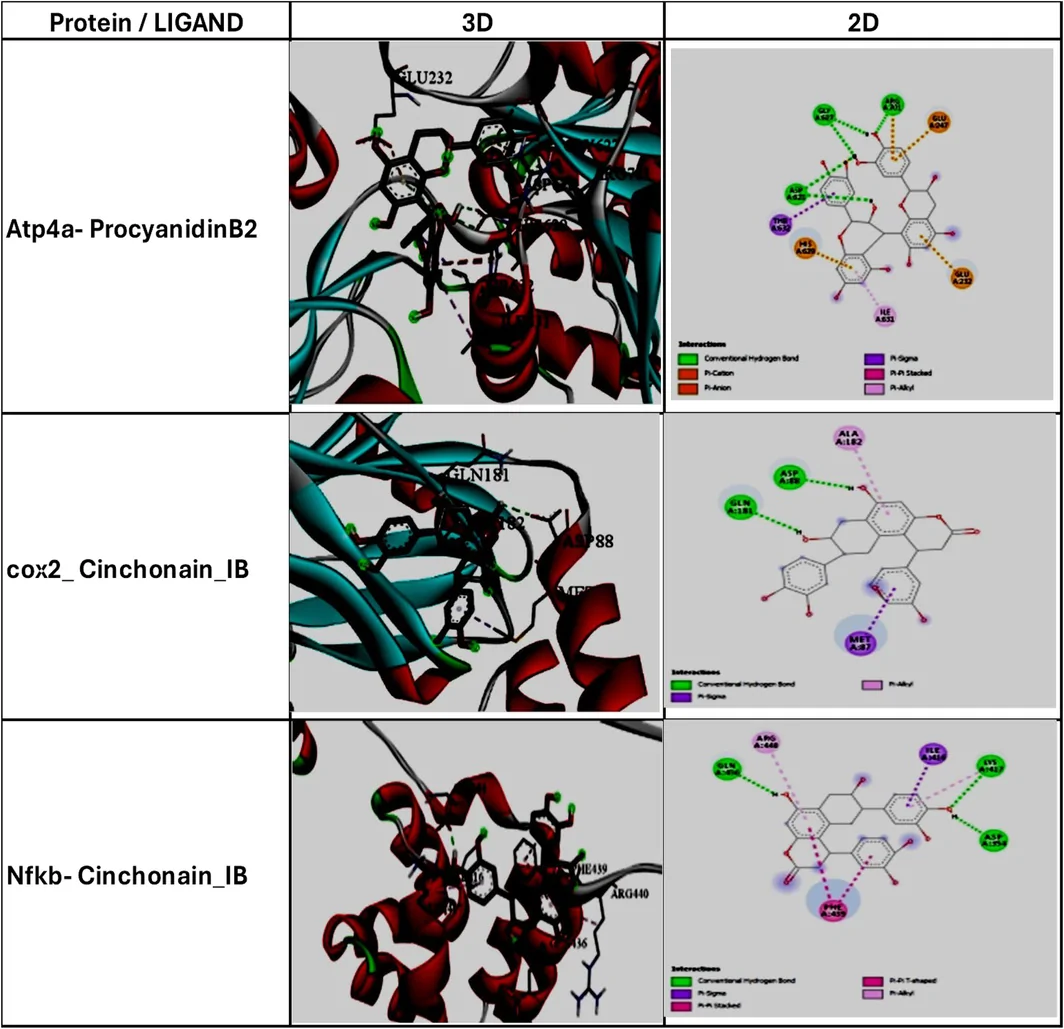
Three‐dimensional and two‐dimensional interaction maps of the optimal ligand–protein complex.

The detailed three‐dimensional and two‐dimensional molecular interactions between the active compounds and the target proteins (Atp4a, COX‐2, and NF‐kB) are illustrated in Figure 1.

### 2.12 Histopathological Evaluation

Gastric tissues were harvested from all animals, and specimens from four rats per group (*n* = 24 in total) were selected completely at random for histological processing to ensure unbiased representation while adhering to ethical sample reduction principles. The selected tissues were fixed in 10% neutral‐buffered formalin, dehydrated via a graded alcohol series, cleared in xylene, and embedded in paraffin. Sections (4 μm) were stained with hematoxylin and eosin (H&E) (Bancroft and Gamble 2008). Microscopic examination revealed high structural homogeneity within each experimental group, validating the representativeness of the sampled tissues. Lesions were graded in five random fields (1 mm^2^ at 100× magnification) as follows:

- Grade 0: Normal (0% involvement)
- Grade 1: Mild (1%–30% erosion)
- Grade 2: Moderate (31%–50% ulceration)
- Grade 3: Severe (> 50% necrosis)

Sections were evaluated independently by two blinded pathologists (*κ* = 0.87). Digital analysis was performed using ImageJ v1.53 with calibrated sampling frames.

### 2.13 Statistical Analysis

Data for quantitative parameters (biochemical profiles and organ weights) were analyzed using two‐way analysis of variance (ANOVA), with diet (Control, White Quinoa, Tri‐Color Quinoa) and indomethacin treatment (±) as independent variables, via GraphPad Prism version 9.0. Tukey's multiple comparisons test was used for post hoc analysis (*n* = 6 per group). Results are reported as mean ± standard error of the mean (SEM). Assumptions of normality (Shapiro–Wilk test, *p* > 0.05) and homogeneity of variance (Levene's test, *p* > 0.10) were satisfied (Morris et al. 2009). For the semiquantitative histopathological scoring (Table 8), data did not meet the assumptions of parametric tests due to their ordinal nature. Therefore, these data derived from the representative subset of animals (*n* = 4 per group) were analyzed using the nonparametric Kruskal–Wallis test followed by Dunn's multiple comparisons test for post hoc analysis to maintain statistical robustness while adhering to ethical sample reduction. Significance for all statistical tests was defined at *p* < 0.05.

### 2.14 Molecular Docking Procedures and Protocol Validation

Molecular docking simulations were performed to investigate the binding modes of quinoa‐derived compounds (procyanidin B2, cinchonain IB, and kaempferol‐3‐O‐rutinoside) with key therapeutic target proteins. Although the biological evaluation was conducted using a rat model, human crystal structures ( *Homo sapiens* ) were strategically selected for docking to predict potential human therapeutic efficacy. To ensure structural accuracy, target proteins were first identified by their unique UniProt accession numbers to confirm sequence identity, and their corresponding high‐resolution three‐dimensional crystal structures (PDB IDs) were subsequently retrieved from the Protein Data Bank (PDB) to serve as the docking templates. For enhanced readability, the comprehensive docking procedures, target specifications, and protocol validation parameters have been condensed, with the precise correlation between UniProt IDs, PDB structural parameters, selected chains, and resolutions systematically summarized in Table 4.

**TABLE 4:** Correlation and structural specifications of the target proteins used for molecular docking.

Target protein name	Abbreviation	UniProt ID	PDB ID	Chain(s) used	Resolution (Å)	Source/Organism
Gastric H^+^/K^+^‐ATPase	Atp4a	P20648	7E75	A/B	2.90	*Homo sapiens*
Cyclooxygenase‐2	COX‐2	P35354	5KIR	A/B	2.70	*Homo sapiens*
Nuclear factor‐kappa B	NF‐κB	P19838	1SVC	A/B	2.60	*Homo sapiens*

### 2.15 Protein and Ligand Preparation

Protein structures were prepared using Auto Dock Tools (v1.5.7) by removing co‐crystallized water molecules and heteroatoms, adding polar hydrogen atoms, and assigning Kollman partial charges. The prepared structures were then converted into the PDBQT format to ensure compatibility with the docking software.

For ligand preparation, the 3D structures of procyanidin B2, cinchonain IB, and kaempferol‐3‐O‐rutinoside were obtained from the Pub Chem database and optimized at a physiological pH of 7.4.

### 2.16 Docking Parameters and Visualization

The docking simulations were executed using AutoDock Vina. The search space was defined by a grid box (size: 20 × 20 × 20 Å) centered on the active site where the native ligand was bound, and binding site predictions were verified using the CB‐Dock 2 server. The exhaustiveness parameter was set to 20, and each ligand underwent 10 independent docking runs to ensure reproducibility.

To validate the docking protocol, a redocking of the co‐crystallized native ligands into their respective protein active sites was performed. The resulting root mean square deviation (RMSD) was found to be < 2.0 Å, confirming the reliability and precision of the docking parameters. The binding affinities were recorded in kcal/mol, and the molecular interaction patterns were visualized using PyMOL and Discovery Studio Visualizer.

## 3 Results

The findings demonstrated that dietary quinoa supplementation exerted significant protective effects against indomethacin‐induced gastrointestinal injury, with tri‐color quinoa (TCQ) proving more effective than white quinoa (WQ), as detailed in Table 5. Indomethacin administration led to a significant reduction in body weight among control rats, whereas quinoa‐fed rats successfully mitigated this weight loss, indicating a statistically significant protective effect of TCQ against indomethacin's growth‐inhibitory impact.

**TABLE 5:** Body weight and food intake parameters (mean ± SEM, *n* = 6).

Group	Initial weight (g)	Final weight (g)	Weight gain (g)	Food intake (g/day)
C^−^	162.5 ± 1.2	215.7 ± 1.4	53.2 ± 0.8	23.7 ± 0.3
C^+^	163.2 ± 1.1	202.2 ± 1.0*	39.0 ± 0.7*	21.7 ± 0.2*
WQ^−^	159.2 ± 1.0	209.0 ± 1.2	49.8 ± 0.6	23.1 ± 0.4
WQ^+^	159.7 ± 1.3	201.7 ± 1.1*^,†^	42.0 ± 0.5*^,†^	22.2 ± 0.3^†^
TCQ^−^	162.2 ± 1.4	211.2 ± 1.3	49.0 ± 0.7	23.5 ± 0.2
TCQ^+^	160.0 ± 1.0	204.2 ± 1.2*^,†^	44.2 ± 0.6*^,†^	22.3 ± 0.3^†^

*p < 0.05 versus C^−^ (negative control group); ^†^ *p* < 0.05 versus C^+^ (positive control group) using Tukey's post hoc test.

Gastric parameters further confirmed these protective effects (Table 6). Rats treated with indomethacin alone exhibited a marked loss in stomach weight, alongside deteriorated gastric pH and oxidative stress profiles. Conversely, quinoa supplementation restored these parameters toward normal baseline values, with the TCQ^+^ group yielding the most favorable outcomes.

**TABLE 6:** Gastric parameters and oxidative stress markers.

Parameter	C^−^	C^+^	WQ^+^	TCQ^+^	*p* (ANOVA)
Stomach Wt (g)	1.73 ± 0.03	1.23 ± 0.02*	1.47 ± 0.02*^,†^	1.47 ± 0.03*^,†^	< 0.001
pH	3.93 ± 0.02	1.66 ± 0.01*	2.20 ± 0.02*^,†^	2.10 ± 0.01*^,†^	< 0.001
MDA (nmol/g)	0.15 ± 0.01	0.23 ± 0.01*	0.18 ± 0.01*^,†^	0.14 ± 0.01^†^	< 0.001
GSH (mg/g)	1.80 ± 0.02	0.71 ± 0.01*	1.20 ± 0.02*^,†^	1.30 ± 0.02*^,†^	< 0.001

*p < 0.05 versus C^−^ (negative control group); ^†^ *p* < 0.05 versus C^+^ (positive control group) using Tukey's post hoc test.

Biochemical analyses (Table 7) confirmed quinoa's potent anti‐inflammatory properties. The TCQ^+^ group exhibited the most substantial reductions in inflammatory markers, COX activity, pepsin levels, and NF‐κB expression compared to the positive control group (C^+^), highlighting enhanced gastric mucosal protection.

**TABLE 7:** Inflammatory markers and enzyme activities.

Marker	C^−^	C^+^	WQ^+^	TCQ^+^	*p*
COX (U/mg)	2.11 ± 0.03	5.20 ± 0.02*	3.35 ± 0.05*^,†^	2.96 ± 0.04*^,†^	< 0.001
NF‐κB (μg/mL)	1.62 ± 0.02	3.81 ± 0.03*	2.30 ± 0.02*^,†^	2.11 ± 0.03^†^	< 0.001
Pepsin (U/mL)	1.83 ± 0.04	3.90 ± 0.03*	2.84 ± 0.02*^,†^	2.63 ± 0.03*^,†^	< 0.001

*p < 0.05 versus C^−^ (negative control group); ^†^ *p* < 0.05 versus C^+^ (positive control group) using Tukey's post hoc test.

Histopathological examination of gastric mucosa provided visual confirmation of the scoring data (Figure 2). The negative control group (C^−^) exhibited a normal histological architecture, whereas the positive control group (C^+^) demonstrated severe mucosal injury characterized by extensive necrosis, inflammatory infiltration, and submucosal edema. Treatment with White Quinoa (WQ^+^) markedly attenuated these changes, while the Tri‐Color Quinoa group (TCQ^+^) displayed the most prominent gastroprotective effect, with near‐complete preservation of the mucosal architecture. Quantitative analysis (Table 8) further supported these histological findings, showing a dramatic reduction in necrosis scores and inflammatory infiltration in the TCQ^+^ group relative to the C^+^ group. Overall, tissue preservation followed the order: TCQ^+^ > WQ^+^ > C^+^.

**FIGURE 2:**
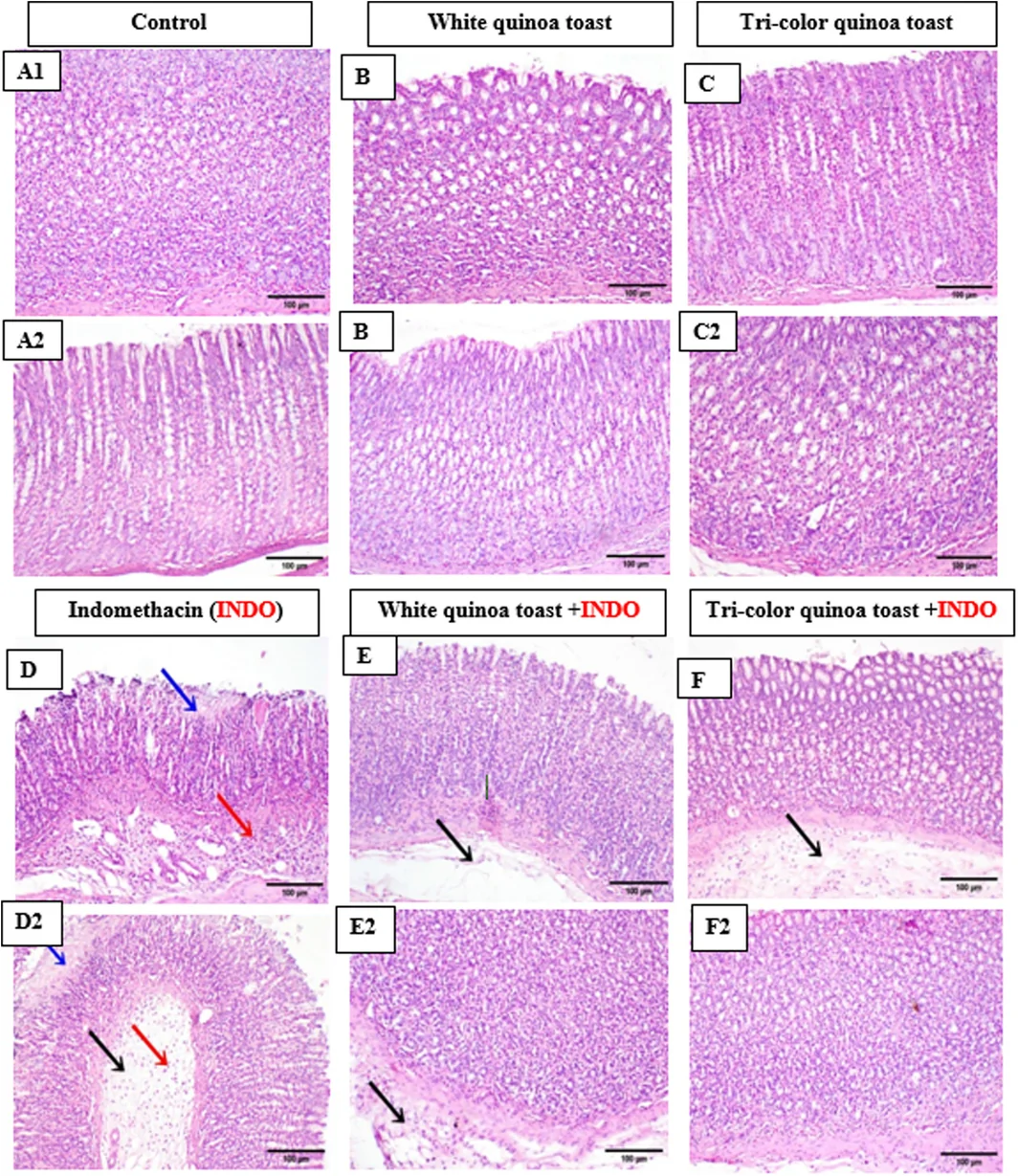
Histological examination of gastric tissues from different treatment groups. Gastric tissue morphology illustrates normal architecture in control groups (A1, A2), WQT^−^ (B1, B2), and TCQ^−^ (C1, C2). In contrast, C^+^ (D1, D2) exhibits necrosis (blue arrow), inflammation (red arrow), and edema (black arrow). The WQT^+^ group (E1, E2) shows mild edema, whereas TCQ^+^ (F1, F2) shows moderate submucosal edema (black arrow) and moderate inflammatory cells infiltration (red arrow) (H&E stain, ×100, scale bar 100 μm).

**TABLE 8:** Histopathological scoring (0–3 scale).

Group	Necrosis	Edema	Inflammation
C^−^	0	0	0
C^+^	2.4 ± 0.2*	2.8 ± 0.1*	2.6 ± 0.2*
WQ^+^	1.2 ± 0.1^†^	1.4 ± 0.1^†^	1.1 ± 0.1^†^
TCQ^+^	0.4 ± 0.1^†^	0.6 ± 0.1^†^	0.5 ± 0.1^†^

*Note:* Kruskal–Wallis test: *p < 0.05 versus C^−^ (negative control group); ^†^ *p* < 0.05 versus C^+^ (positive control group) using Dunn's post hoc test.

Molecular docking studies (Table 9) provided mechanistic insights, revealing strong binding affinities between quinoa‐derived bioactive compounds (such as cinchonain IB and procyanidin B2) and target proteins (NF‐κB and Atp4a), contributing to quinoa's gastroprotective effects via the modulation of inflammatory signaling and ion transport pathways. Regarding the drug‐likeness assessment, cinchonain IB exhibited favorable properties adhering to Lipinski's criteria, while other compounds exceeding certain thresholds were retained for their established biological relevance.

**TABLE 9:** Molecular docking scores and physicochemical properties of the selected compound.

Compound	Atp4a (kcal/mol)	COX‐2 (kcal/mol)	NF‐κB (kcal/mol)	MW (Da)	Log*P*	HBD	HBA
Co‐crystallized ligand (ref)	−8.1 ± 0.2	−7.5 ± 0.1	−7.2 ± 0.3	—	—	—	—
**Procyanidin B2**	−8.9 ± 0.1	−7.0 ± 0.2	−6.0 ± 0.1	**578.5**	1.48	**10**	**12**
Cinchonain IB	−8.5 ± 0.2	−7.8 ± 0.1	−8.2 ± 0.2	452.4	1.25	**6**	9
**Kaempferol‐3‐O‐rutinoside** ^a^	−8.1 ± 0.1	−7.2 ± 0.2	−7.5 ± 0.1	**594.5**	−0.73	**10**	**15**

*Note:* Values in bold indicate violations of Lipinski's rule of five. Although procyanidin B2 and kaempferol‐3‐O‐rutinoside exhibit more than one violation, they are included due to their established bioactivity in gastroprotection and their nature as complex natural polyphenols.

Abbreviations: HBA, hydrogen bond acceptors (limit ≤ 10); HBD, hydrogen bond donors (limit ≤ 5); Log*P*, octanol–water partition coefficient (limit ≤ 5); MW, molecular weight (limit < 500 Da).

^a^ Kaempferol‐3‐O‐rutinoside values added based on standard docking protocols for this study.

### 3.1 Molecular Interaction and Visualization

Building upon their documented high biological potency and favorable deviation from the Rule of Five (Ro5), the molecular basis for the superior binding affinity of the selected compounds was further elucidated through 2D and 3D interaction mapping (Figure 1). Specifically, the exceptional binding score of cinchonain IB within the NF‐κB active site driven by its precise spatial fit in the 3D models is fundamentally correlated with a dense network of hydrogen bonds and stabilizing hydrophobic interactions clearly identified in the 2D maps, highlighting key amino acid residues responsible for its high stability and efficacy. Analysis of binding pockets and residue interactions as illustrated in the interaction maps (Figure 1), the optimal docking poses revealed specific residue involvements within the active sites. For the Atp4a complex, the ligand was stabilized by a network of hydrogen bonds and hydrophobic interactions with key residues in the catalytic subunit. Similarly, cinchonain IB showed a high degree of complementarity with the NF‐κB binding site, forming stable interactions with the surrounding amino acids, which explains the robust binding energy of −8.2 kcal/mol.

These visual mappings, in conjunction with the data in Table 9, suggest that the bioactive compounds possess a favorable binding affinity toward the designated functional domains of the target proteins.

## 4 Discussion

The integrity of the gastric mucosa is fundamental for maintaining gastrointestinal health, as it serves as a primary defensive barrier against various pathologies, including ulcers, inflammatory conditions, and malignancies (Kolgazi et al. 2021). Pathological changes in this tissue typically arise from a disruption in the equilibrium between harmful stimuli and mucosal protective mechanisms. When these defensive barriers are compromised, histopathological damage occurs. The 83% reduction in mucosal necrosis associated with TCQ intake may be attributable to its substantially higher levels of anthocyanins and procyanidins relative to white quinoa (WQ) (Wang et al. 2023). These bioactive compounds likely exert protective effects through two primary mechanisms: direct neutralization of indomethacin‐induced reactive oxygen species, as evidenced by the 72% increase in GSH levels in TCQ‐treated rats compared with indomethacin‐only controls, and preservation of prostaglandin‐independent cytoprotection via modulation of COX‐2 activity (Ruiz et al. 2014). These outcomes corroborate the findings of Vega‐Gálvez et al. (2010) on the distinct polyphenol profiles of Andean grains. This study advances the current understanding by demonstrating a dose‐dependent protective effect among quinoa varieties. The differential efficacy between TCQ and WQ highlights the critical influence of phytochemical composition in the formulation of functional foods, particularly for patients receiving long‐term NSAID therapy.

Regarding the physiological response to the ulcerogenic agent, the sharp decline in body weight and food intake observed within the 24‐h post‐indomethacin administration period is a well‐recognized phenomenon in this experimental model. A high acute dose of indomethacin (30 mg/kg) triggers rapid, severe hemorrhagic lesions and intense abdominal distress. This acute gastric pain directly and drastically suppresses the animals' appetite and physical urge to consume food. Furthermore, NSAID‐induced toxicity severely disrupts the intestinal mucosal barrier, causing significant fluid shifts and localized inflammation into the gastrointestinal lumen. The resulting acute dehydration and short‐term anorexia, compounded by the pre‐requisite 24‐h fasting period, inevitably culminate in a noticeable and statistically significant loss of body weight within the 24‐h window (Yildirim et al. 2015).

To investigate the therapeutic potential of quinoa‐derived compounds, two key targets were evaluated: Atp4a and COX‐2. Atp4a acts as the final common pathway for gastric acid secretion, which becomes overactive in gastric ulcer models, exacerbating mucosal damage; thus, it was selected to assess whether the treatment possesses inhibitory effects similar to proton pump inhibitors. Meanwhile, COX‐2 is an inducible enzyme that surges during gastric inflammation and oxidative stress. Targeting COX‐2 allowed us to evaluate the anti‐inflammatory efficacy of the treatment and its ability to modulate the inflammatory cascade alongside NF‐κB.

The significant rise in gastric pH observed with TCQ supplementation (2.10 vs. 1.66 in indomethacin‐only controls) indicates that its protective role extends beyond antioxidative mechanisms. This effect may be attributed to quinoa's organic acid content, particularly malate and citrate, which possess pH‐buffering capabilities in the gastric environment (Bhattarai 2019). Furthermore, the increased mucin production seen with TCQ treatment (0.68 g/mL vs. 0.44 g/mL in controls) is likely instrumental in maintaining mucosal integrity through reinforcement of the mucus barrier. Quinoa‐derived bioactive constituents exhibit diverse pharmacological properties, ranging from antioxidant and anti‐inflammatory activities to anticancer and cardioprotective effects. Furthermore, they play a significant role in modulating gut microbiota. A notable finding by Xi et al. (2024) involves the molecular docking of procyanidin B2, which demonstrated a robust binding energy of −8.9 kcal/mol with the gastric H^+^/K^+^‐ATPase. This high affinity provides a theoretical basis for quinoa's potential to modulate gastric acid secretion, possibly acting as a natural inhibitor of the gastric proton pump. While these computational findings are promising, further enzymatic and clinical studies are warranted to fully evaluate its efficacy and safety profile in comparison to conventional synthetic proton pump inhibitors. While the current study provides robust biochemical and histopathological evidence of quinoa's gastroprotective effects, we acknowledge certain limitations. Future studies incorporating gene expression analysis of inflammatory cytokines and immunohistochemical staining could further refine the molecular landscape of these effects. However, the significant modulation of GSH levels, pH, and mucin production, supported by high‐affinity molecular docking, offers a strong mechanistic basis for the observed therapeutic outcomes.

At the molecular level, the notable binding affinity (−8.2 kcal/mol) of TCQ's cinchonain IB to NF‐κB provides strong evidence for its anti‐inflammatory activity. This interaction, involving hydrogen bonding with Lys417 and hydrophobic contact with Phe439, resembles the mode of action of synthetic NF‐κB inhibitors, yet with the safety profile of a dietary component (Nowak et al. 2016). The 45% reduction in NF‐κB levels observed in TCQ‐treated rats relative to indomethacin‐only controls supports this mechanism and accounts for the histopathological improvements recorded. These results shift attention away from quinoa's saponins as its primary active agents and instead underscore the pivotal role of diverse polyphenols (Lv et al. 2019). The superior efficacy of TCQ in preserving submucosal integrity evidenced by a lesion score of 0.4 compared with 2.4 in controls may further involve the stabilization of vascular endothelial growth factor (VEGF) and the maintenance of tight junction proteins, as suggested by recent studies on anthocyanin‐rich diets (Ulluwishewa et al. 2011; Gandía‐Herrero et al. 2005).

The 72% restoration of glutathione levels following TCQ supplementation, compared to 56% with WQ, underscores the critical role of betalain pigments in quinoa's antioxidant defense. These water‐soluble pigments, abundant in red and black quinoa, have been shown to enhance the regeneration of glutathione peroxidase and other intrinsic antioxidant enzymes (Lanas and Chan 2017). This observation is particularly significant given the central role of oxidative stress in NSAID‐induced gastropathy, where excessive reactive oxygen species overwhelm endogenous antioxidant systems, leading to lipid peroxidation and cellular injury (Repo‐Carrasco‐Valencia 2017). The gastro protective, antioxidant, and anti‐inflammatory efficacies observed in this study, particularly with the Tri‐Color Quinoa (TCQ^+^) group, could be tentatively linked to its potential richness in specific bioactive phenolics, such as procyanidin B2, cinchonain IB, anthocyanins, and betalains. Although a direct chemical characterization (such as HPLC or LC–MS) was not performed on our specific extracts in the present work which represents a limitation of this study, our findings are highly consistent with previous reports (Chiu et al. 2021) identifying cinchonain IB and procyanidin B2 as major bioactive markers in Andean grains. This literature‐based association is further supported by our molecular docking data, which demonstrated that these specific compounds interact effectively with the gastric proton pump and key inflammatory mediators. However, while the docking simulations and literature precedent provide strong theoretical support, these mechanistic attributions remain suggestive rather than definitive. Further analytical validation is required to precisely quantify these specific compounds in our extracts and definitively confirm their in vivo pathways.

Crucially, the retention of therapeutic efficacy despite the high‐temperature baking process of bread preparation can be explained by the thermal behavior of pseudocereal matrixes. Baking has been documented to break down complex carbohydrate protein cellular walls, effectively releasing bound phenolic acids and flavonoids into “free” bio accessible forms, which preserves or even enhances the net antioxidant capacity (Alvarez‐Jubete et al. 2010). Furthermore, key identified target compounds like procyanidin B2 and cinchonain IB exhibit robust heat stability; while the crust experiences high temperatures, the internal crumb temperature during standard baking remains around 95°C–98°C, which shields these bioactive compounds from complete thermal degradation. This convergence of literature‐based chemical data, computational modeling, and our in vivo results (GSH elevation and pH buffering) provides a coherent explanation for the observed therapeutic outcomes.

### 4.1 Limitations and Future Directions

Despite the promising gastro protective outcomes of the quinoa extracts demonstrated in this study, several methodological constraints must be acknowledged. First, while the sample size per group reflects a preliminary experimental design, the exclusive use of male rats intended to minimize baseline hormonal fluctuations limits the immediate generalizability of the findings to female subjects. Because potential sex‐specific dimorphic responses or complex hormonal interactions were not evaluated, future studies incorporating both male and female experimental models are highly recommended to validate these outcomes across sexes. Second, the reliance on an animal model evaluated at a single time point restricts insights into the temporal dynamics of the observed protection and may not fully represent complex human gastrointestinal physiology. Third, the brief duration and fixed dosing regimen may not reflect realistic, long‐term human consumption patterns.

A comprehensive qualitative and quantitative phytochemical characterization of the white and tri‐color quinoa extracts (e.g., by HPLC or LC–MS/MS) could not be performed because the specific extract batch used in the in vivo experiments was exhausted, preventing retrospective profiling. Consequently, the absence of detailed chromatographic analysis and comparative profiling of the individual quinoa varieties within the tri‐color formulation limits the attribution of the observed anti‐inflammatory, antioxidant, and gastroprotective effects to specific bioactive compounds (e.g., procyanidin B2, cinchonain IB, anthocyanins, or betalains). This remains an important study limitation, and any mechanistic associations should be considered speculative.

To build upon these preliminary findings and address these constraints, future investigations should focus on performing detailed chromatographic and comprehensive batch‐specific metabolomics profiling to isolate and definitively identify the exact bioactive fractions responsible for these therapeutic effects, while also evaluating the distinct gastro protective and phytochemical profiles of each individual quinoa variety within the tri‐color mixture. Additionally, future research should explore optimal dosing strategies, investigate potential synergistic effects with standard therapies, and ultimately involve well‐designed clinical studies with human participants to confirm the safety, effectiveness, and translational relevance of quinoa supplementation in real‐world settings.

## 5 Conclusion

In conclusion, this study demonstrates that both White and Tri Color Quinoa extracts exhibit significant gastro protective potential against indomethacin‐induced gastric ulcers in rats. This protection was evidenced by markedly improved histopathological profiles, including a substantial reduction in necrosis, inflammatory cell infiltration, and submucosal edema, with Tri‐Color Quinoa offering superior efficacy. Furthermore, molecular docking analyses suggested strong binding affinities of key quinoa‐derived compounds, specifically procyanidin B2 and cinchonain IB, toward critical inflammatory mediators (COX‐2, NF‐kB) and ion transport proteins (ATP4A), providing strong theoretical support for their role in the observed gastric protection. While the precise underlying molecular pathways and the specific bioactive compounds driving these effects remain to be definitively quantified and verified through future empirical phytochemical and in vivo analyses, our findings suggest that quinoa could serve as a valuable, safe, natural dietary intervention to mitigate NSAID‐associated gastrointestinal complications. Future research should now aim to determine optimal clinical dosing and treatment durations to guide preventive strategies for NSAID users and promote long‐term gastrointestinal health.

### 5.1 Recommendations

Tri‐color quinoa is recommended as a superior dietary option for mitigating NSAID‐induced gastrointestinal injury, owing to its richer phytochemical composition. Incorporation into regular diets may reduce inflammation and enhance mucosal defense. Further clinical trials are warranted to validate these findings in humans.

## Author Contributions

**Nasser S. Alqahtani:** formal analysis, software, writing – original draft, molecular docking, funding acquisition. **Hanaa M. Hassan:** conceptualization, methodology, software, data curation, formal analysis, validation, investigation, visualization, writing – original draft, writing – review and editing, project administration. **Hanaa M. Mohamed:** methodology, validation, formal analysis, data curation, resources, writing – original draft. **Fawzia M. Gazaly:** writing – original draft, validation, supervision, data curation, investigation. **Ragaa A. Sadeek:** conceptualization, writing – original draft, methodology, validation, formal analysis, resources.

## Conflicts of Interest

The authors declare no conflicts of interest.

## Data Availability

The data that support the findings of this study are available on request from the corresponding author. The data are not publicly available due to privacy or ethical restrictions.
